# Comparison of Four Tourmalines for PS Activation to Degrade Sulfamethazine: Efficiency, Kinetics and Mechanisms

**DOI:** 10.3390/ijerph19063244

**Published:** 2022-03-09

**Authors:** Yongli Jiao, Ying Zhang, Wei Wang

**Affiliations:** Tianjin Key Laboratory of Environmental Technology for Complex Trans-Media Pollution, College of Environmental Science and Engineering, Nankai University, Tianjin 300350, China; jyl@nankai.edu.cn (Y.J.); zhangying04@nankai.edu.cn (Y.Z.)

**Keywords:** tourmaline, persulfate, sulfamethazine, electron-hole, ^1^O_2_

## Abstract

Four types of tourmalines (TMs, S1, S2, S3 and S4) for activating persulfate (PS) to degrade sulfamethazine (SMT) were compared to find the most efficient catalyst. The four TMs were mesoporous materials with abundant functional groups, but were different in terms of size, composition, specific surface area, contact angle, and zero potential point. The removal of SMT in S1, S2, S3 and S4 systems with PS at the optimum reaction conditions ([SMT]_0_ = 5 mg/L, [PS]_0_ = 4 mM, [TM]_0_ = 5 g/L, pH_0_ = 5, and T = 25 °C) were 99.0%, 25.5%, 26.0%, and 51.0%, respectively, which might be related to the metal content of TM. Although the degradation of SMT in the S1/PS/SMT system was not dominated by SO_4_^•−^ and •OH, the radicals contributed to the SMT removal in the S2, S3, and S4 systems. ^1^O_2_ and holes both contributed to the degradation of SMT in the four systems. The metal at the X position might be related to the generation of ^1^O_2_ and holes, while Fe of TM was mainly related to the generation of free radicals, such as SO_4_^•−^. Electrochemical impedance spectroscopy tests confirmed that the separation of electrons and holes on the TM surface could be promoted by adding PS and SMT. S1 presented a higher electron-transfer rate than the other three TMs. The PS activation by TM with a high metal content at the X position provided an efficient and low-consumption treatment for antibiotic refractory wastewater.

## 1. Introduction

Tourmaline (TM) is a kind of annular silicate mineral with boron as the characteristic element. It is mainly found in granite pegmatite, gas hydrothermal deposits, and metamorphic rocks [1]. According to different characteristics, it can be divided into iron TM, alkali TM, and magnesium TM [2,3]. The general formula of TM is XY_3_Z_6_T_6_O_18_(BO_3_)_3_V_3_W where X represents Na^+^, Ca^2+^, K^+^, or a vacancy and Y denotes Fe^2+^, Fe^3+^, Al^3+^, etc. [4,5]. The dissociation of Na and Ca leaves electrons at the X position. The electric neutrality of TM is maintained by trapping holes, resulting in the formation of an electron–hole structure, which agrees with vacancy formation in NiO [6,7].

The characteristics of TM, such as the abundant mineral components and sufficient surface-active sites, enable it to behave as a catalyst. Being similar to NiO, TM was proved to be able to activate persulfate (PS) to degrade contaminants through a non-radical pathway in our previous study [8]. TM is a common, economical, and eco-friendly natural mineral with complex structure and chemical composition. The activation of PS by TM can overcome the disadvantages of the other PS activation methods, such as high cost and poor practicability [9,10]. Therefore, the TM/PS process deserves comprehensive and in-depth study. However, very little research has been done in this area. Our previous studies on the activation of PS by TM have revealed part of the reaction mechanisms [8]; however, some mechanisms of the process, such as the influence of the metal at X position and the iron content on activating PS, remain unclear.

TMs from different areas have different compositions and properties. TM in China is mainly distributed in Xinjiang, Inner Mongolia, Yunnan, Tibet, and other places. In this study, four kinds of TMs with different metal contents from different produces of origin were selected and denoted as S1, S2, S3 and S4. Among them, S1, S3, and S4 were produced in different mining areas of Xinjiang, and S2 was produced in Hunan. The TM used in our previous research was S1 [8]. Sulfamethazine (SMT) as a common sulfonamide antibiotic has been commonly used in livestock production and widely exists in the environment [11,12,13]. It is used as the target contaminant in this study. This research has been conducted to compare the performance of four TMs in activating PS to degrade SMT, investigate the influence of TM component on PS activation, and further disclose the reaction mechanisms of the TM/PS process.

## 2. Materials and Methods

### 2.1. Materials

In this study, the four types of tourmalines (TMs) were commercial production provided by Tianjin Hongyan Tianshan Stone Industry Co., Ltd. (Tianjin, China). All used reagents were of analytical grade and were used without further purification. Potassium persulfate, methanol, L-histidine and Nafion solution were purchased from Meryer (Shanghai) Chemical Technology Co., Ltd. (Shanghai, China), Tianjin Concord Technology Co., Ltd. (Tianjin, China), Tianjin Solomon Biotechnology Co., Ltd. (Tianjin, China) and Qingdao Tenglong Microwave Technology Co., Ltd. (Qingdao, China), respectively. NaOH and Na_2_C_2_O_4_ were purchased from Tianjin Jiangtian Chemical Technology Co., Ltd. (Tianjin, China). The pH of the solution was adjusted with NaOH and HCl (from Tianjin Chemical Reagents Factory 5) solution.

### 2.2. Experimental Procedures

The degradation of SMT (5 mg L^−1^) was conducted in a 100 mL Erlenmeyer flask at 25 ± 1 °C. The flask was put in a Thermostatic Water Bath with a rotating speed set at 180 r/min. TM was added to the flask to initiate the reaction. The pH of the working solution was adjusted by the addition of 0.1 M NaOH and 0.1 M HCl solutions. Samples were taken at selected time intervals and an appropriate amount of methanol to quench the reaction was added. Samples taken from the flask were filtered with 0.22 µm membrane to remove the TM particles. The filtered samples were stored in brown vials, refrigerated at 4 °C, and measured within 24 h.

### 2.3. Materials Characterization and Sample Analysis

Scanning electron microscopy (SEM, JSM-7800F, JEOL, Tokyo, Japan), X-ray diffraction (XRD-7000, Shimadzu, Kyoto, Japan), and Fourier transform infrared spectroscopy (FT-IR, Nicolet IS50, Thermofisher, Waltham, MA, USA) were used to provide information regarding the surface and composition of TMs. A surface area and porosimetry analyzer (Micromeritics ASAP2460, Micromeritics, Norcross, GA, USA) was used to provide information of the pore size and volume and the specific surface area of the TM samples. The contact angles of TMs were measured using a contact angle meter (JC200DM, China). Zero-potential points were measured according to the approaches described by Srivastava et al. [14]. Details of the measurement can be referred to from Text S1 (Zero-potential point measurement) in the Supporting Information (SI). Electrochemical impedance spectroscopy (EIS) analysis was conducted with an electrochemical workstation. FTO coated with TM, saturated calomel electrode, and a platinum electrode were used as the working electrode, reference electrode and counter electrode, respectively. The preparation of the working electrode was conducted following the method (Text S2 Working electrode preparation) proposed by Wang et al. [15]. The concentration of SMT was measured using a high-performance liquid chromatograph (Ultimate 3000, Thermofisher, Waltham, MA, USA) with a C18 column (Details in Text S3 SMT measurement procedure).

## 3. Results and Discussion

### 3.1. TM Characterization

#### 3.1.1. SEM and EDS

**Figure 1:**
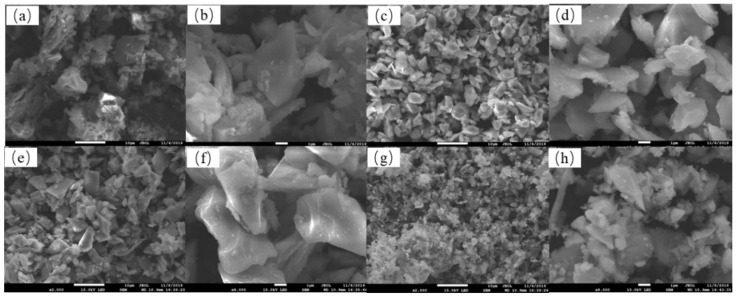
SEM images of S1 (**a**) ×2000 and (**b**) ×8000, S2 (**c**) ×2000 and (**d**) ×8000, S3 (**e**) ×2000 and (**f**) ×8000 and S4 (**g**) ×2000 and (**h**) ×8000. (**a**,**b**) were reproduced from Ref. [8] with permission from Elsevier.

The SEM images of four TM samples were provided in Figure 1.

The results of the S1 system have been presented by Zhang et al. [8]. In this system, PS promoted the electron–hole separation on TM and contaminants were degraded mainly through a non-radical pathway [13,16]. In contrast, in the AgVO_3_/PS system hydroxyl radicals and sulfate radicals were reactive oxygen species in the degradation of organic contaminants. The presence of AgVO_3_ greatly promoted the decomposition of peroxodisulfate and produced a large number of free radicals [17,18]. We quoted the results of the S1 system from the article for comparison (with permission from Elsevier). All of samples were irregular in shape and aggregated. This aggregation might be caused by the spontaneous polarization and attractive force of the particles [19]. The particle size of S2 was more evenly distributed than that of S1. Small particles of S2 attached to the surface of large particles. The SEM image of S3 was similar to that of S2. The particles of S3 attached to each other and there was accumulation among the particles. S4 had the smallest size, the particles were interdependent, and there was “chain connection” between small particles.

EDS was used to analyze the element content of four TM samples (Table S1). S1 contained O (52.2%), Na (1.6%), Mg (6.3%), Si (11.3%), Al (8.0%) and Ca (20.6%) [8]. S2 contained O (45.3%), Na (1.6%), Si (18.5%), Al (16.6%) and Fe (18.0%). S3 had O (46.4%), Na (1.6%), Si (21.9%), Al (15.0%), and Fe (15.1%). S4 contained O (53.9%), Na (0.8%), Si (11.9%), Al (9.2%), Mg (5.2%), Ca (5.3%), Fe (6.4%), Ti (3.8%) and P (3.5%). The element distribution and content on the surface of tourmaline would affect the element detection by EDS. Therefore, the elements with low content were not detected, such as B. The amount of Fe followed the order of S2 > S3 > S4 > S1. Fe on TM surface might be the active sites for PS decomposition, however, the amount of Fe did not agree with the SMT removal in four systems. This indicated that iron content was not determinant for the contaminant degradation in the TM/PS process. The amount of Na and Ca on the X sites of TM followed the order of S1 > S4 > S2 = S3, which was in line with the SMT removal, indicating that these two elements might be related to contaminant removal. The dissociation of the metal at the X position induced the generation of electron–hole structure, which enhanced the yield of ^1^O_2_ through the oxidation of O_2_^•−^ or O_2_. The holes could also directly degrade SMT. Therefore, the higher the amount of metal at X position, the faster the removal of contaminant might be.

#### 3.1.2. XRD

The XRD of TMs is given in Figure S1 and the images indicated the crystal structure of the four catalysts. The component of TM was greatly influenced by its place of origin. In addition to the characteristic peaks of standard TM, the XRD of S1 also included the peaks of AlSiO_5_, SiO_2_, Ca_2_MgSi_2_O_7_, Na_2_Si_2_O_5_, Na_6_(AlSiO_4_)_6_, etc. S2 and S3 included the peaks of SiO_2_, and S4 included the peaks of TiO_2_ and Ca_2_MgSi_2_O_7_. The presence of the peaks of calcium compounds in XRD agreed with the EDS results.

#### 3.1.3. FTIR

**Figure 2:**
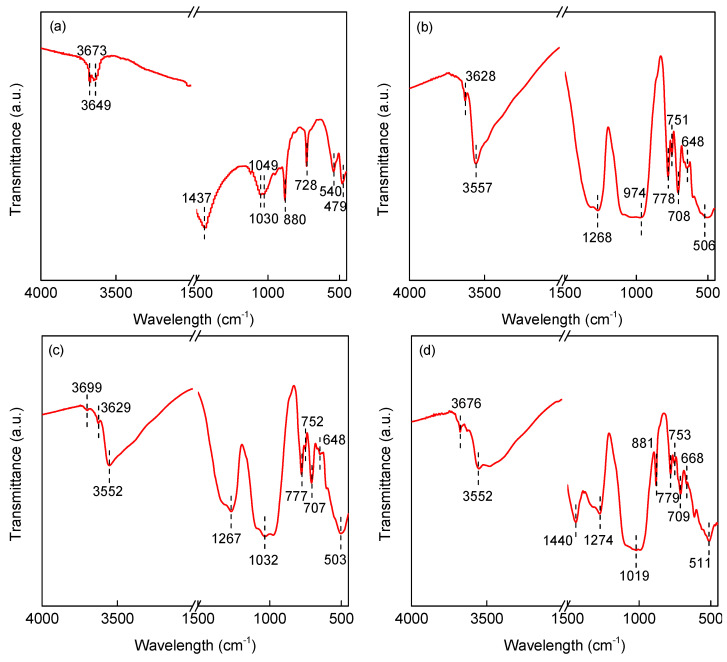
FTIR images of (**a**) S1, (**b**) S2, (**c**) S3 and (**d**) S4. (**a**) was reproduced from Ref. [8] with permission from Elsevier.

The FTIR spectra of TM shown in Figure 2 presented the peaks of the vibration of silica tetrahedron, [BO_3_] triangle, surface hydroxyl group and a triple octahedral cation.

The vibration of silica tetrahedron included v_as_(Si-O-Si), v_s_(Si-O-Si), v_as_(O-Si-O), v_s_(O-Si-O), Si(Al)-O and δ(Si-O). The surface hydroxyl vibration was commonly distributed in the 1600–3700 cm^−1^ region, such as 3649 cm^−1^ and 3673 cm^−1^ for S1, 3628 cm^−1^ and 3557 cm^−1^ for S2, 3699 cm^−1^, 3629 cm^−1^ and 3552 cm^−1^ for S3, and 3676 cm^−1^ and 3552 cm^−1^ for S4. The [BO_3_] triangle vibration was generally located in the region of 1200–1450 cm^−1^, such as 1437 cm^−1^ for S1, 1268 cm^−1^ for S2, 1267 cm^−1^ for S3, and 1274 cm^−1^ for S4. Silicon-oxygen tetrahedron oscillations generally occurred in the region of 400–1200 cm^−1^. The type and position of vibration were related to the structure of TM. The v(Si(Al)-O) peak was located at 1030 cm^−1^ and 880 cm^−1^ for S1, 974 cm^−1^, 778 cm^−1^ and 751 cm^−1^ for S2, 1032 cm^−1^, 777 cm^−1^ and 752 cm^−1^ for S3, and 1019 cm^−1^, 881 cm^−1^, 779 cm^−1^ and 753 cm^−1^ for S4. The peak position of v_as_(O-Si-O) of S1 was 1049 cm^−1^. No such peaks were observed in the FTIR images of S2, S3, and S4. The positions for v(Si-O-(Al) Si) peak were 728 cm^−1^ and 540 cm^−1^ for S1, 708 cm^−1^ and 648 cm^−1^ for S2 and S3, and 709 cm^−1^ and 668 cm^−1^ for S4. The peaks of δ(Si-O) were at 479 cm^−1^ for S1 and S2, 503 cm^−1^ for S3, and 511 cm^−1^ for S4.

#### 3.1.4. BET

**Figure 3:**
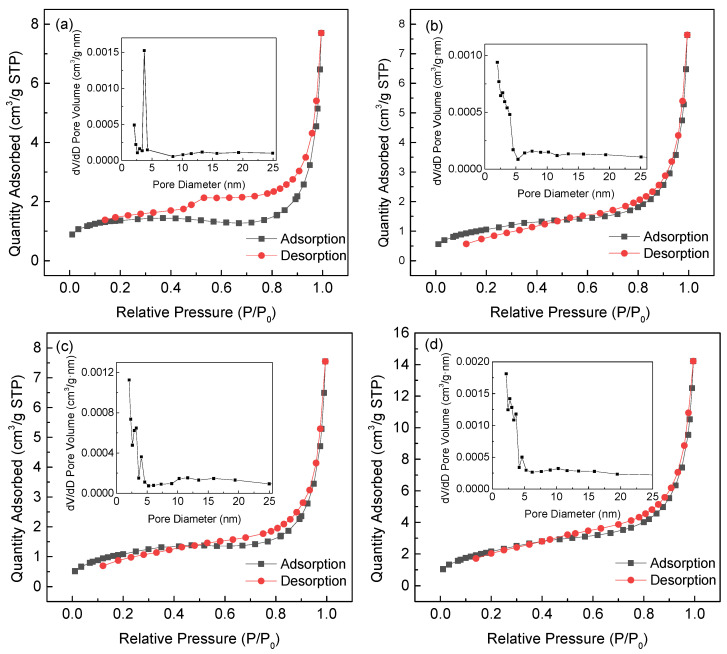
N_2_ adsorption–desorption isotherms and pore size distribution of (**a**) S1, (**b**) S2, (**c**) S3 and (**d**) S4. (**a**) was reproduced from Ref. [8] with permission from Elsevier.

Figure 3 presented the N_2_ adsorption–desorption isotherms and pore structure distribution of the four TMs.

Table S2 showed the surface area and pore parameters of four TMs. According to the IUPAC classification, the N_2_ adsorption–desorption isotherms showed the characteristics of type IV isotherms, with obvious adsorption hysteresis in the pressure regions of 0.2–0.99 P/P_0_ (S1), 0.5–0.99 P/P_0_ (S2), 0.5–0.99 P/P_0_ (S3), and 0.4–0.99 P/P_0_ (S4), belonging to type H3 [20,21]. Moreover, there was no adsorption termination platform in the high-pressure region of the four samples, indicating that the pore structure was irregular and there were still large pores in the catalysts. Compared with S1, S2, S3 and S4 had wider pore size distributions. The specific surface areas, pore volumes, and average aperture were 5.10 m^2^/g, 0.0100 cm^3^/g and 14.3 nm, respectively, for S1; 3.85 m^2^/g, 0.0110 cm^3^/g and 11.3 nm, respectively, for S2; 3.58 m^2^/g, 0.0110 cm^3^/g and 12.4 nm, respectively, for S3; and 6.99 m^2^/g, 0.021 cm^3^/g and 11.2 nm, respectively, for S4. The pore size of the TMs was mainly below 5 nm (the insets of Figure 3), indicating the TMs used in this study were mesoporous materials.

#### 3.1.5. Contact Angles

**Figure 4:**
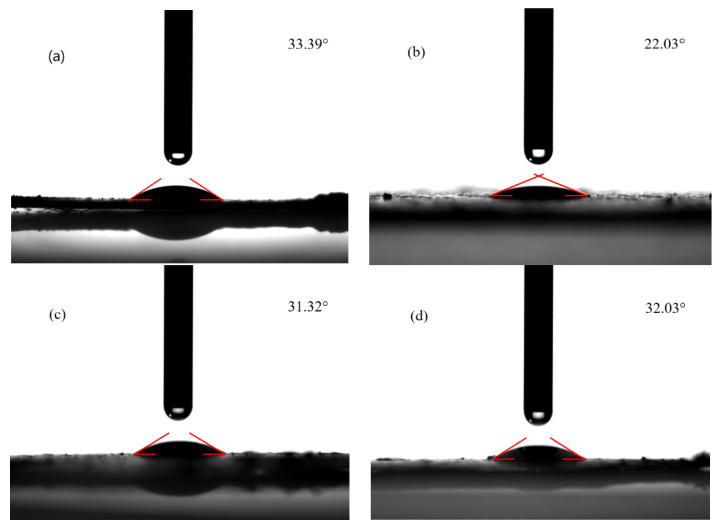
Contact angles of (**a**) S1, (**b**) S2, (**c**) S3 and (**d**) S4. (**a**) was reproduced from Ref. [8] with permission from Elsevier.

The contact angle (θ) was used to characterize the hydrophilicity of the material surface. The solid surface is hydrophilic at θ < 90°, indicating that the liquid is more likely to wet the solid. The surface of the solid is hydrophobic at θ > 90°, indicating that the liquid can easily move on the surface. As shown in Figure 4, the contact angles of four TMs were 33.39° (S1), 22.03° (S2), 31.32° (S3), and 32.03° (S4), indicating that the four samples were hydrophilic and could contact well with the PS solution, which was beneficial to the interface reaction. The electric field of TMs could change the structure of water cluster by changing the hydrogen bond network arrangement [22,23,24], which strengthened the hydrogen bond between the water and TM.

#### 3.1.6. Zero-Potential Point

In order to compare the electric properties of four TM surfaces and analyze their charged properties in the reaction system, the zero-potential points of the samples were determined. As shown in Figure S2, pH_zpc, S1_ = 8.4, pH_zpc, S2_ = 8.0, pH_zpc, S3_ = 6.7, and pH_zpc, S4_ = 8.0. At pH < pHzpc, the TM surface was positively charged, which was conducive to contact with S_2_O_8_^2−^. At pH > pH_zpc_, the TM surface was negatively charged and had electrostatic repulsion to S_2_O_8_^2−^. The pH_zpc_ of S1 was higher than that of S2, S3, and S4, indicating that S1 had a wider positive charge range and could have better contact with the negative S_2_O_8_^2−^.

### 3.2. Comparison of Four TMs

**Figure 5:**
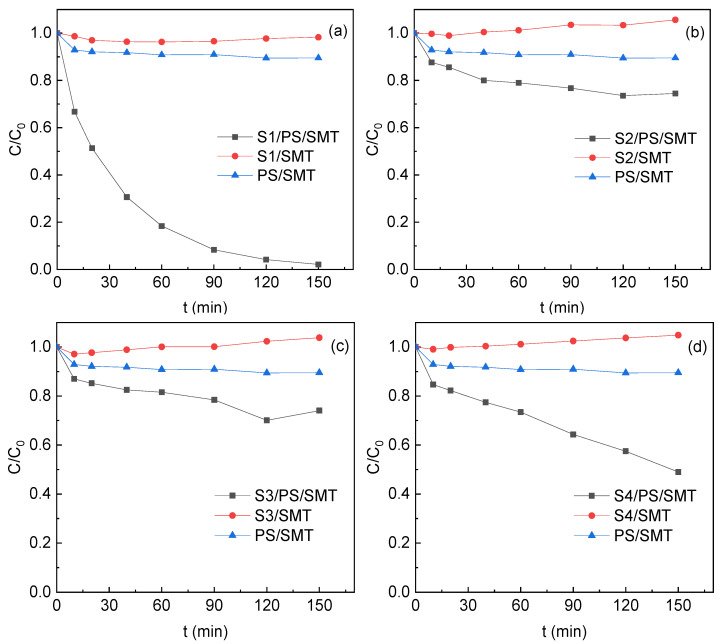
SMT degradation in PS activation by (**a**) S1, (**b**) S2, (**c**) S3 and (**d**) S4. Reaction conditions: [SMT]_0_ = 5 mg/L, [PS]_0_ = 4 mM, [TM]_0_ = 5 g/L, pH_0_ = 5 and T = 25 °C. (**a**) was drawn with modification after Zhang et al. [8].

The efficiency of four TMs in activating PS to degrade SMT was compared and presented in Figure 5.

The removal of SMT within 150 min by PS oxidation only was around 10%, and there was no adsorption removal of SMT by four TMs. The SMT removal increased with the reaction time in S1/PS, S2/PS, S3/PS and S4/PS systems. The removal of SMT at 150 min in the S1, S2, S3 and S4 systems was 99.0%, 25.5%, 26.0% and 51.0%, respectively. The removal followed the decreasing order of S1 > S4 > S2 ≈ S3, in line with the amount of Na and Ca on the X sites of TMs. S1 with the most abundant Na and Ca presented the highest SMT removal. The results indicated that the metal at the X position might have an important effect on the PS activation and degradation of SMT. As depicted in Section 3.4, Na and Ca of TMs dissociated in water and formed “electron–holes”. The holes and electrons were separated effectively with the presence of PS and SMT. ^1^O_2_ was generated by the transformation of O_2_^•−^ produced by the reduction of O_2_ with electrons or the oxidation of O_2_ by holes [25,26]. Both ^1^O_2_ and holes contributed to the SMT degradation.

### 3.3. The Effect of pH

TM is the only mineral in nature with spontaneous polarity and has the ability to regulate the solution pH. Therefore, the ability of four TMs to regulate the solution pH was compared (Figure S3). When the initial pH was equal to 2, the presence of S1, S2, S3, and S4 increased the final pH to 8.1, 6.8, 3.0, and 8.0, respectively. At initial pH 5, the final pH of the S1, S2, S3 and S4 systems was 8.4, 8.1, 6.4 and 8.1, respectively. At initial pH 10, the final pH of the S1, S2, S3 and S4 systems was 8.3, 8.3, 7.8, and 8.3, respectively. The pH regulation ability of samples S1 and S4 were higher than that of S2 and S3, which was in line with the SMT removal for the four systems.

The ability of tourmaline to adjust the pH of acidic solution was mainly derived from two aspects [27,28]: (i) the spontaneous permanent electrical polarity of TM. The electric field on the surface of TM particles could electrolyze water to produce H_2_ and hydrated hydroxyl ions, which increased the solution pH. (ii) The surface properties of TM. There were a lot of hydroxyl groups and metal suspended bonds on the surface of the crushed TM particles, which could adsorb or replace H^+^ in aqueous solution, increasing the solution pH. It could be found from the EDS and BET analysis that compared with S1 and S4, S2 and S3 samples had relatively smaller specific surface area and lower content of Na and Ca elements on the surface and, thus, fewer sites that could adsorb or replace H^+^. Therefore, the final pH of the S2 and S3 systems was stabilized at acid or neutral due to that the two TMs had weak ability to adjust the pH of acidic solution. S1 and S4 with higher content of Na and Ca adjusted the solution pH to alkaline. The pH decrease of the alkaline solution was primarily due to the fact that the metal on the TM surface was likely to adsorb OH^−^, resulting in surface hydroxylation and solution pH decrease. Thus, the solution pH could be adjusted within a short time after TM was added. CO_2_ in the air reached an equilibrium at the gas–liquid interface as the reaction proceeded, resulting in the stabilization of the solution pH. The ability of TM to adjust the solution pH was also affected by the size and dosage of tourmaline, as well as the stirring conditions.

**Figure 6:**
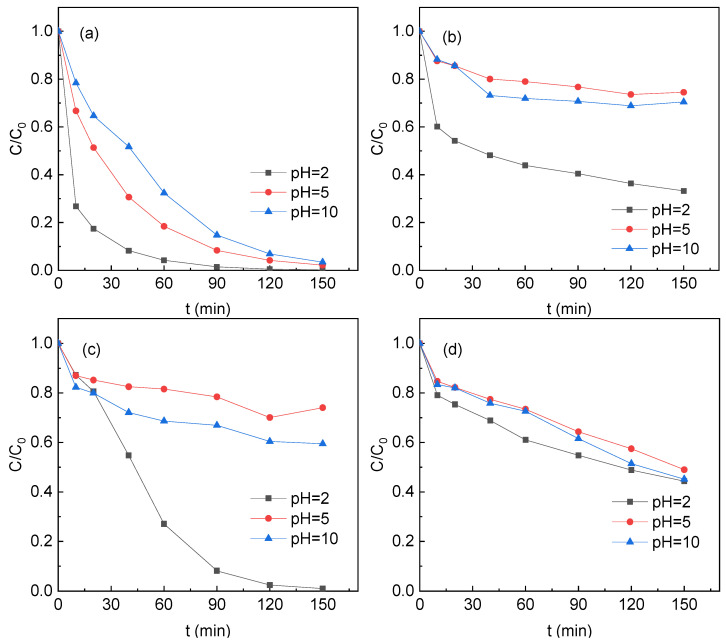
SMT removal at different pH (**a**) S1, (**b**) S2, (**c**) S3 and (**d**) S4. Reaction conditions: [SMT]_0_ = 5 mg/L, [PS]_0_ = 4 mM, [TM]_0_ = 5 g/L, and T = 25 °C. (**a**) was drawn with modification after Zhang et al. [8].

The effect of pH on the efficiency of PS activation by TM was investigated and the results were provided in Figure 6.

At an initial pH of 2, the final removal of SMT in the S1, S2, S3, and S4 systems was 99.8%, 66.8%, 99.0% and 55.7%, respectively. At an initial pH 5, the final removal of SMT in the S1, S2, S3, and S4 systems was 99.0%, 25.5%, 26.0%, and 51.0%, respectively. At an initial pH 10, the final removal of SMT in the S1, S2, S3, and S4 systems was 96.5%, 29.6%, 40.6% and 54.8%, respectively. The removal of SMT in the S2, S3, and S4 systems at pH_0_ = 10 was slightly higher than that at pH_0_ = 5. At pH_0_ = 10, the spontaneous polarization of TM maintained the solution pH at alkali. S2, S3, and S4 contained Fe, which could activate PS to produce SO_4_^•^^−^ and this radical could be converted into •OH under alkaline conditions, promoting the degradation of SMT in the system.

Under acidic conditions, the removal of SMT in the S1 system was greatly improved, which might be due to the protonation of tourmaline surface, being conducive to the contact of S_2_O_8_^2−^ with TM. The enhanced SMT removal at pH = 2 in the S2, S3 and S4 systems might be due to the Fe content of the particles and the solution pH variation. The Fe content in the three samples followed a decreasing order of S4 > S2 > S3. At initial pH 2, the solution pH was maintained at around 8.0 (S4), 6.8 (S2), and 3.0 (S3). The acidic pH of the S2 and S3 systems might promote the release of iron to the solution, which could accelerate the activation of PS to produce more SO_4_^•^^−^ for SMT degradation. The high solution pH of the S4 systems hindered Fe dissolution during the reaction and thus PS activation. In order to confirm the hypothesis, methanol was used to scavenge SO_4_^•^^−^ and •OH at pH = 2. The SMT removal in the S2, S3, and S4 systems was hindered by the presence of methanol (Figure S4). The results confirmed the hypothesis that, for the S2, S3, and S4 systems, the acidic condition promoted the Fe dissolution and thus PS activation to produce more SO_4_^•^^−^. It could be also concluded that compared with the iron content, the pH variation during the reaction was more important to the SMT removal in the TM/PS systems.

### 3.4. Reactive Species

**Figure 7:**
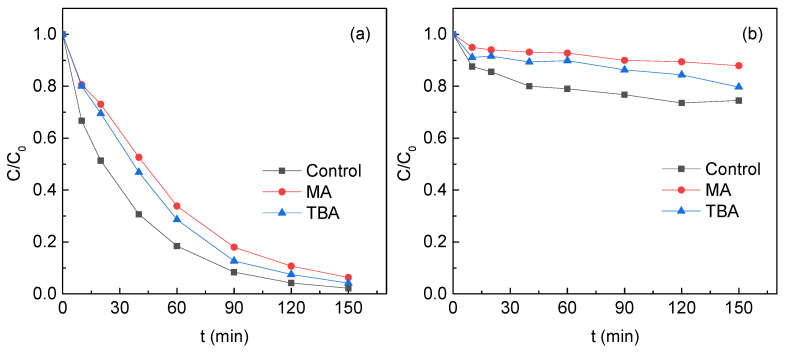

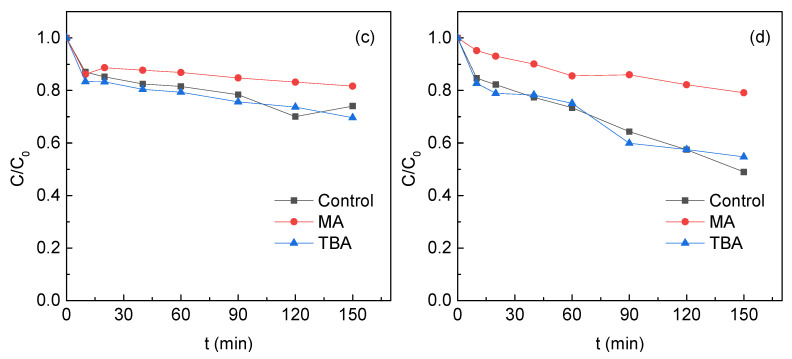
MA and TBA influence on SMT removal in PS activation by (**a**) S1, (**b**) S2, (**c**) S3 and (**d**) S4. Reaction conditions: [SMT]_0_ = 5 mg/L, [PS]_0_ = 4 mM, [TM]_0_ = 5 g/L, [MA]_0_ = [TBA]_0_ = 50 mM, pH_0_ = 5 and T = 25 °C. (**a**) was drawn with modification after Zhang et al. [8].

In this study, we used radical quenching method to identify the role of free radicals. Methanol (MA) and tert-butyl alcohol (TBA) were used to investigate the radicals generated in the TM/PS systems. The reaction rate constant between MA and •OH was 9.7 × 10^8^ M^−1^s^−1^ and 3.8–7.6 × 10^8^ M^−1^s^−1^ for the reaction of TBA and •OH. The reaction rate constant was 1.0 × 10^7^ M^−1^s^−1^ for MA and SO_4_^•^^−^ and 4–9.1 × 10^5^ M^−1^s^−1^ for TBA and SO_4_^•^^−^ [29,30]. Therefore, MA could be used to scavenge both •OH and SO_4_^•^^−^, while TBA was used to only scavenge •OH. As shown in Figure 7, MA and TBA did not significantly inhibited the degradation of SMT in the S1 system, indicating that neither SO_4_^•^^−^ nor •OH was the main species for SMT removal.

The addition of TBA and MA in the S2 system decreased the SMT removal at 150 min from 25.5% to 20.3% and 12.0%, respectively, indicating that SO_4_^•^^−^ and •OH played almost an equal role in the SMT removal. The SMT removal in the S3 and S4 system in the presence of MA decreased by 7.70% and 30.1%, respectively. However, TBA did not show any inhibiting effect on the SMT removal in the S3 and S4 systems. The results indicated that SO_4_^•^^−^ was the only radical for SMT removal in these two systems at the experimental conditions.

**Figure 8:**
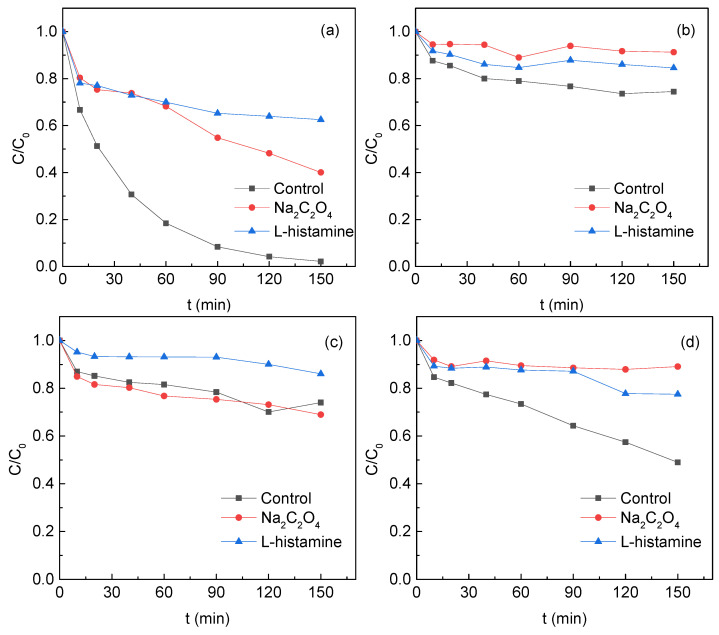
Influence of Na_2_C_2_O_4_ and L-histamine on SMT removal (**a**) S1, (**b**) S2, (**c**) S3 and (**d**) S4. Reaction conditions: [SMT]_0_ = 5 mg/L, [PS]_0_ = 4 mM, [TM]_0_ = 5 g/L, pH_0_ = 5 and T = 25 °C. (**a**) was drawn with modification after Zhang et al. [8].

L-histidine and sodium oxalate were used to capture ^1^O_2_ and holes on the TM surface, respectively (Figure 8).

For the S1 system, SMT removal in the presence of L-histidine and sodium oxalate was 37.4% and 59.9%, respectively, being much lower than that in the control experiment (97.8%). The SMT removal in the S2 system with added L-histidine and sodium oxalate decreased by 10.1% and 16.8%, respectively. For the S3 system, the addition of Na_2_C_2_O_4_ did not impose any negative influence on the SMT removal, suggesting that holes on the TM surface did not affect the contaminant removal. The addition of L-histidine to the S3 system decreased the SMT removal by 12%. For the S4 system, the presence of L-histidine and sodium oxalate decreased the SMT removal from 51.0% to 10.9% and 22.5%, respectively. These results indicated that, unlike S3, both ^1^O_2_ and holes might play important roles in the SMT degradation in the S1, S2, and S4 systems. The surface metal (Na and Ca) of TMs dissociated in water and formed the most “electron-holes”. When PS and SMT coexisted in the systems, the holes and electrons could be separated effectively. Oxygen captured electrons to generate O_2_^•−^, which could be directly recombined to generate ^1^O_2_ to degrade SMT. Holes could oxidize O_2_ to generate ^1^O_2_ [25,26].

### 3.5. Electrochemical Test

**Figure 9:**
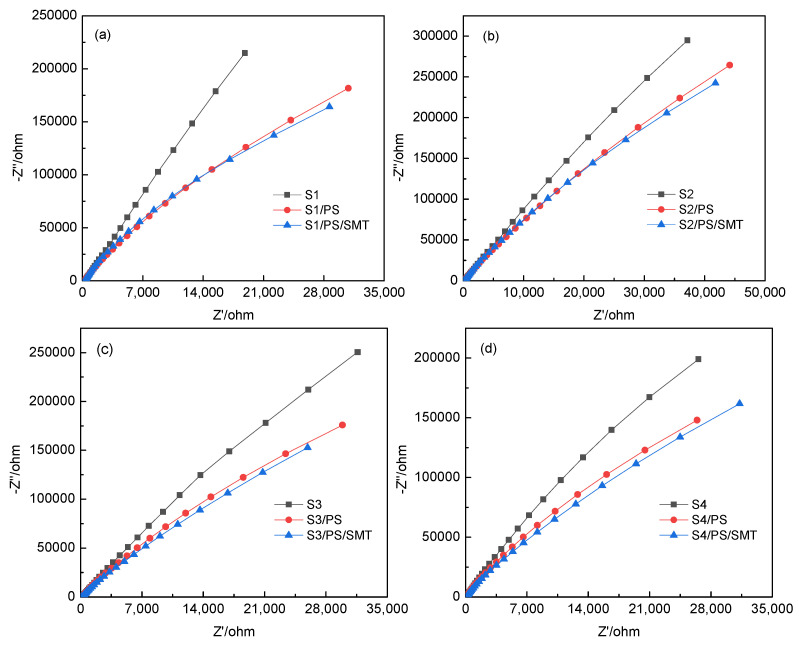
EIS images of (**a**) S1, (**b**) S2, (**c**) S3 and (**d**) S4. Reaction conditions: [SMT]_0_ = 5 mg/L, [PS]_0_ = 4 mM, [TM]_0_ = 5 g/L, pH_0_ = 5 and T = 25 °C. (**a**) was reproduced from Ref. [8] with permission from Elsevier.

EIS was conducted to investigate the charge transfer resistance of the four TMs (Figure 9).

The smaller the arc radius in Nyquist plot is, the smaller the charge transfer resistance will be. This will result in faster charge transfer rate and thus a more effective separation of holes and electrons on the TM surface. Compared with TM alone, the Nyquist arc of TM in the presence of PS and SMT was smaller, indicating that the addition of PS and SMT promoted the charge transfer. Compared with that of S2, S3, and S4, the Nyquist arc radius of S1 with the addition of PS and SMT decreased the most, indicating the highest efficiency of the separation of holes and electrons, which agreed with the metal content on the S1 surface. This confirmed that the formation of “electron–holes” in the TM/PS/SMT system was related to the dissociation of Na and Ca ions on the TM surface.

### 3.6. Reaction Mechanisms

S1 was produced in Xinjiang and contained the least amount of iron. The reaction mechanism of the S1/PS system can be found by referring to our previous research [8].

S2 sample was produced in Hunan Province, and its iron content was the highest among the four samples. The SMT degradation mechanism of S2/PS/SMT system confirmed by the scavenging experiment and electrochemical test was proposed in three pathways: (i) the surface metal (Na and Ca) of TMs dissociated in water and formed “electron–holes”. The holes on the TM surface directly oxidize some SMT. (ii) Fe on the TM surface activate PS to generate SO_4_^•^^−^ that then transformed to •OH. Both SO_4_^•^^−^ and •OH contributed to the SMT degradation. However, because of the pH adjusting ability of TM, the solution pH of the S2 and S4 systems was maintained at neutral or alkaline conditions for most pHs investigated. This was not beneficial to the PS activation by the Fe on the TM surface. (iii) ^1^O_2_ participated in the SMT degradation and its mechanism was the same as that of the S1/PS/SMT system. Briefly, O_2_ captured electrons to produce O_2_^•−^ that then transformed to ^1^O_2_. The oxidation of O_2_/O_2_^•−^ also yielded ^1^O_2_. SO_4_^•^^−^, •OH, ^1^O_2_ and holes all contributed to the SMT removal. Although the Fe content in S2 was higher than in S1, the SMT removal in the S2 system was lower than that in the S1 system, which might be due to the low content of Na and Ca at the X position.

Both S3 and S4 samples were produced in Xinjiang, ranking second and third in terms of iron content, respectively. The SMT degradation mechanisms of the S3 and S4 systems were similar to that of the S2 system. However, the radical responsible for the degradation of SMT in these two systems was SO_4_^•^^−^ rather than •OH. Therefore, SO_4_^•^^−^, ^1^O_2_ and holes were the main species to remove SMT in these two systems.

## 4. Conclusions

Four TMs were investigated in terms of their ability to activate PS to degrade SMT. The contact angles indicated that the four TMs were all hydrophilic and could contact well with the aqueous solution, being beneficial to the real wastewater treatment. The pH_zpc_ of S1 indicated that S1 had a wider positive charge range and could have better contact with negative S_2_O_8_^2−^. The SMT removal in the four TM/PS systems followed the decreasing order of S1 > S4 > S2 ≈ S3, which was in line with the Na and Ca content in the TMs, indicating the contaminant removal was related to the content of the two metals. The amount of Fe in the TMs followed the order of S2 > S3 > S4 > S1, which would affect the production of SO_4_^•^^−^ and •OH. For S2, S3, and S4, the release of Fe to the solution was promoted at the acidic condition, which enhanced the activation of PS to produce more SO_4_^•^^−^ for SMT degradation. The scavenging experiments proved that SO_4_^•^^−^ and •OH together with ^1^O_2_ and holes contributed to the SMT removal in the S2/PS systems. SO_4_^•^^−^, ^1^O_2_ and holes were the main species for the SMT removal in the S3/PS and S4/PS systems. The activity of TM was mainly affected by the metal contents at the X position and the Fe content on TM. Metal at the X position determined the non-radical degradation pathway of SMT, while Fe was related to the generation of free radicals, the yield of which was affected by the solution pH. EIS confirmed a faster electron transfer rate in the S1/PS system compared with the other three systems, which might be due to the dissociation of high content of Na and Ca. TM as a green, common, and cheap catalyst might be used in catalyzing oxidants to treat real wastewater.

## Data Availability

Not applicable.

## Supplementary Materials

The following supporting information can be downloaded at: https://www.mdpi.com/article/10.3390/ijerph19063244/s1, Text S1: Zero-potential point measurement, Text S2: Working electrode preparation, and Text S3: SMT measurement procedure; Figure S1: XRD images of (a) S1, (b) S2, (c) S3 and (d) S4; Figure S2: pHZPC of S1, S2, S3 and S4; Figure S3: pH changes during PS activation by (a) S1, (b) S2, (c) S3 and (d) S4. Reaction conditions: [SMT]_0_ = 5 mg/L, [PS]_0_ = 4 mM, [TM]_0_ = 5 g/L and T = 25 °C; Figure S4: MA influence on SMT removal in PS activation by (a) S1, (b) S2, (c) S3 and (d) S4 at pH 2. Reaction conditions: [SMT]_0_ = 5 mg/L, [PS]_0_ = 4 mM, [TM]_0_ = 5 g/L, [MA]_0_ = 50 mM, pH = 2 and T = 25 °C; Table S1: The element content of TM (%); Table S2: Surface area and pore parameters of four tourmalines.
