# Supplementary material for: Comparison of Four Tourmalines for PS Activation to Degrade Sulfamethazine: Efficiency, Kinetics and Mechanisms
Source: Int J Environ Res Public Health. 2022 Mar 9;19(6):3244. doi: 10.3390/ijerph19063244 (PMC8951620; doi:10.3390/ijerph19063244)
Supplement: Supplementary file 1 [file ijerph-19-03244-s001.zip › ijerph-1568213-supplementary.pdf]

SI for

Comparison of four tourmalines for PS activation to degrade sulfamethazine: Efficiency, kinetics and mechanisms

Yongli Jiao, Ying Zhang, Wei Wang*

Tianjin Key Laboratory of Environmental Technology for Complex Trans-Media Pollution, College of Environmental Science and Engineering, Nankai University, Tianjin 300350, China

* Corresponding authors at: College of Environmental Science and Engineering, Nankai University, Tianjin 300350, China. *E-mail address*: nkwangwei@126.com (W. Wang).

Text S1 Zero-potential point measurement

For the measurement of zero-potential points, 0.01 mol/L NaCl solution was prepared and the pH of the solution was adjusted using NaOH and HCl to 2-11. 0.2 g TM was added into 50 mL NaCl solution with different pH and the mixture was vibrated in a thermostatic oscillator for 48 h. Then, the final pH of the solution was measured. The point of intersection of initial pHs and final pHs was the zero-potential point of TM.

Text S2 Working electrode preparation

In the EIS analysis, FTO coated with TM was used as the working electrode. To preparation of the working electrode, TM particles were added into Nafion solution, and the mixture was treated by water bath sonication (30 min) and vortexing (30 min) to ensure uniform particle dispersion. Then 20 μL TM/Nafion suspension was coated onto FTO, which was dried naturally at room temperature to form the working electrode.

Text S3 SMT measurement procedure

SMT was analyzed using a high-performance liquid chromatograph (Ultimate 3000, USA) with an Agilent ZORBAX SB C18 column (column temperature = 30 ℃). The mobile phase was acetonitrile/water at a ratio of 35:65 (% V/V) and the flow rate was 0.5 mL/min. The signal of SMT was detected at 270 nm.

Figure captions:

Figure S1 XRD images of (a) S1, (b) S2, (c) S3 and (d) S4.

Figure S2 pH_{ZPC} of S1, S2, S3 and S4.

Figure S3 pH changes during PS activation by (a) S1, (b) S2, (c) S3 and (d) S4. Reaction conditions: $[SMT]_0$ = 5 mg/L, $[PS]_0$ = 4 mM, $[TM]_0$ = 5 g/L and T = 25°C.

Figure S4 MA influence on SMT removal in PS activation by (a) S1, (b) S2, (c) S3 and (d) S4 at pH 2. Reaction conditions: $[SMT]_0$ = 5 mg/L, $[PS]_0$ = 4 mM, $[TM]_0$ = 5 g/L, $[MA]_0$ = 50 mM, pH = 2 and T = 25°C.

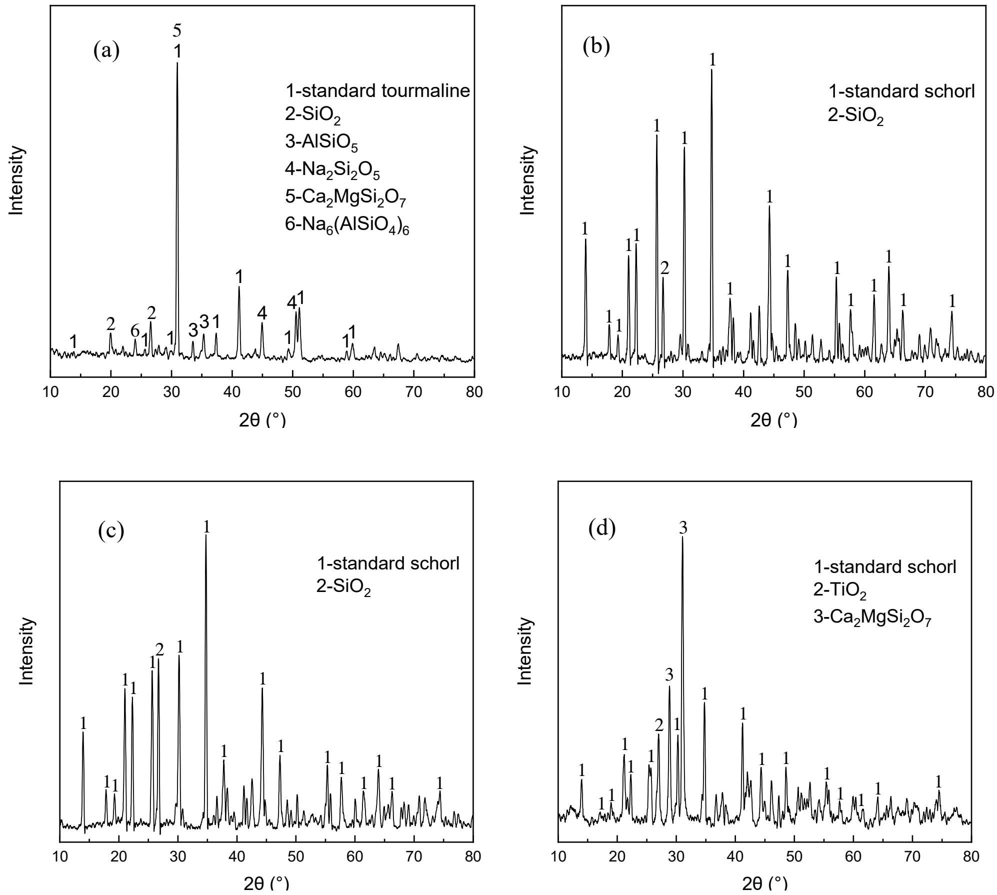

Figure S1. XRD images of (a) S1, (b) S2, (c) S3 and (d) S4.

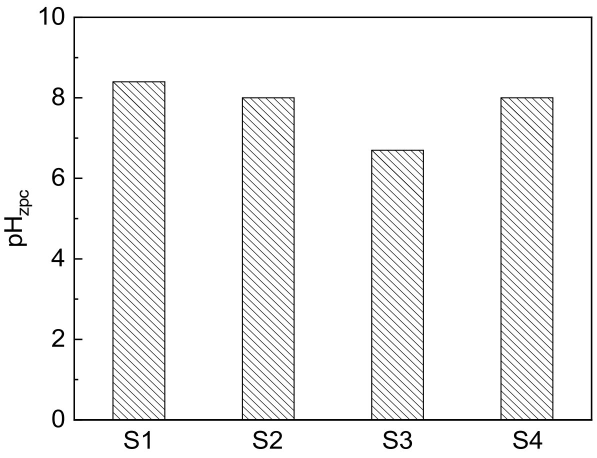

Figure S2. pH_{ZPC} of S1, S2, S3 and S4.

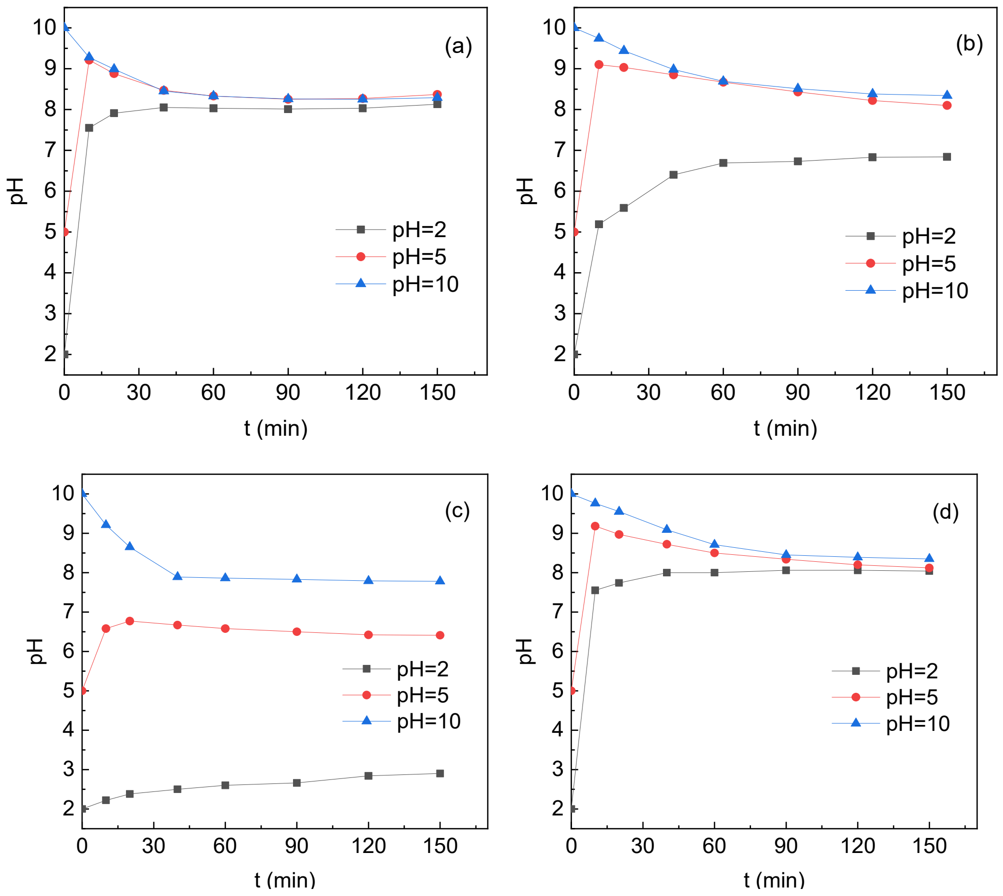

Figure S3. pH changes during PS activation by (a) S1, (b) S2, (c) S3 and (d) S4. Reaction conditions: $[SMT]_0$ = 5 mg/L, $[PS]_0$ = 4 mM, $[TM]_0$ = 5 g/L and T = 25°C.

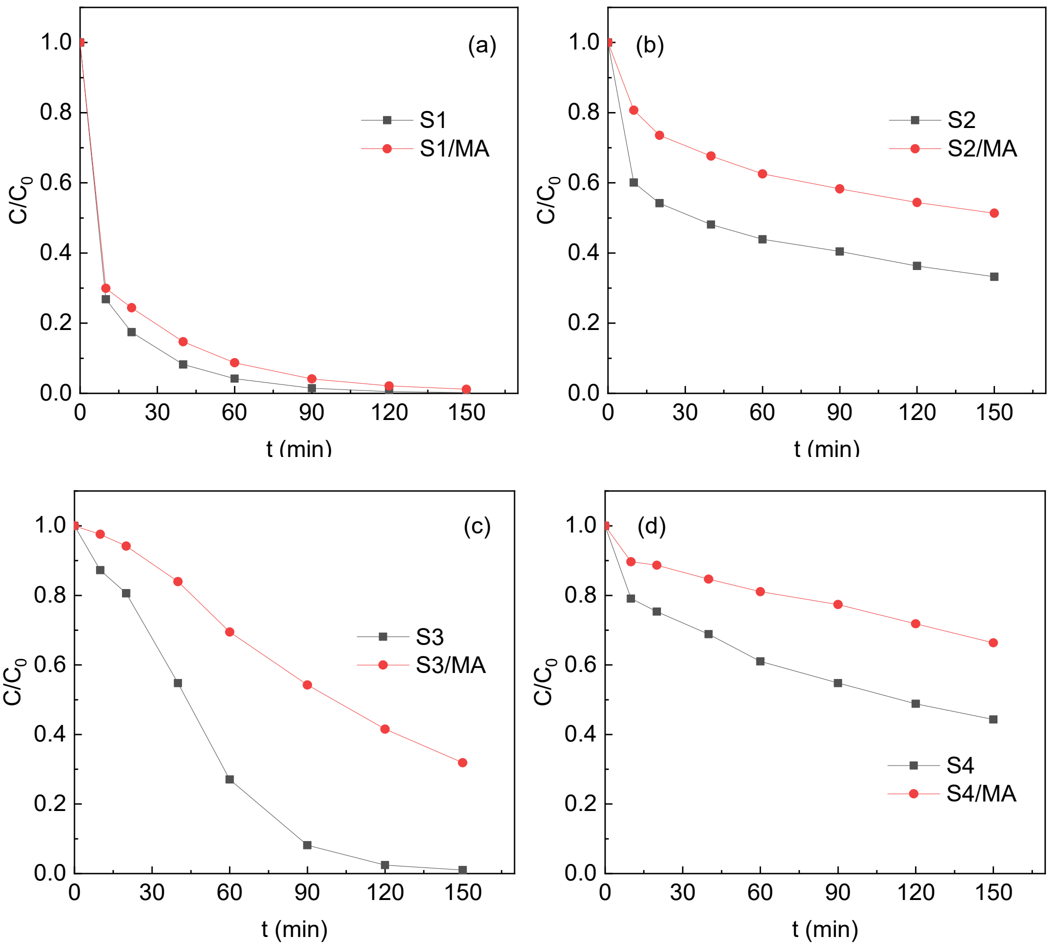

Figure S4. MA influence on SMT removal in PS activation by (a) S1, (b) S2, (c) S3 and (d) S4 at pH 2. Reaction conditions: $[SMT]_0$ = 5 mg/L, $[PS]_0$ = 4 mM, $[TM]_0$ = 5 g/L, $[MA]_0$ = 50 mM, pH = 2 and T = 25°C.

Table S1 The element content of TM (%).

Sample	O	Si	Al	Mg	Na	Ca	Fe	Ti	P	totle
S1	52.2	11.3	8.0	6.3	1.6	20.6	0	0	0	100.0
S2	45.3	18.5	16.6	0	1.6	0	18.0	0	0	100.0
S3	46.4	21.9	15.0	0	1.6	0	15.1	0	0	100.0
S4	53.9	11.9	9.2	5.2	0.8	5.3	6.4	3.8	3.5	100.0

Table S2 Surface area and pore parameters of four tourmalines.

Sample	S_{BET} (m^2/g)	V_p (cm^3/g)	D_p (nm)
S1	5.098	0.010	14.255
S2	3.850	0.011	11.276
S3	3.579	0.011	12.402
S4	6.991	0.021	11.223
